# Evolution of biofilm-forming pathogenic bacteria in the presence of nanoparticles and antibiotic: adaptation phenomena and cross-resistance

**DOI:** 10.1186/s12951-021-01027-8

**Published:** 2021-09-27

**Authors:** Riti Mann, Amy Holmes, Oliver McNeilly, Rosalia Cavaliere, Georgios A. Sotiriou, Scott A. Rice, Cindy Gunawan

**Affiliations:** 1 The iThree Institute, University of Technology Sydney, Ultimo, NSW 2007 Australia; 2 School of Pharmacy and Medical Sciences, The University of South Australia, Adelaide, Australia; 3 Department of Microbiology, Tumor and Cell Biology, Karolinska Institutet, Stockholm, Sweden; 4 Singapore Centre for Environmental Life Sciences Engineering, Singapore, Singapore; 5 School of Biological Sciences, Nanyang Technological University, Singapore, Singapore; 6 School of Chemical Engineering, University of New South Wales, Sydney, NSW 2052 Australia

**Keywords:** Nanoparticle, Antibiotic resistance, *Pseudomonas*, Pathogen, Biofilm, Persistence

## Abstract

**Background:**

Treatment of bacterial biofilms are difficult and in many cases, expensive. Bacterial biofilms are naturally more resilient to antimicrobial agents than their free-living planktonic counterparts, rendering the community growth harder to control. The present work described the risks of long-term use of an important alternative antimicrobial, silver nanoparticles (NAg), for the first time, on the dominant mode of bacterial growth.

**Results:**

NAg could inhibit the formation as well as eradicating an already grown biofilm of *Pseudomonas aeruginosa*, a pathogen notorious for its resilience to antibiotics. The biofilm-forming bacterium however, evolved a reduced sensitivity to the nanoparticle. Evidence suggests that survival is linked to the development of persister cells within the population. A similar adaptation was also seen upon prolonged exposures to ionic silver (Ag^+^). The persister population resumed normal growth after subsequent passage in the absence of silver, highlighting the potential risks of recurrent infections with long-term NAg (and Ag^+^) treatments of biofilm growth. The present study further observed a potential silver/antibiotic cross-resistance, whereby NAg (as well as Ag^+^) could not eradicate an already growing gentamicin-resistant *P. aeruginosa* biofilm. The phenomena is thought to result from the hindered biofilm penetration of the silver species. In contrast, both silver formulations inhibited biofilm formation of the resistant strain, presenting a promising avenue for the control of biofilm-forming antibiotic-resistant bacteria.

**Conclusion:**

The findings signify the importance to study the nanoparticle adaptation phenomena in the biofilm mode of bacterial growth, which are apparently unique to those already reported with the planktonic growth counterparts. This work sets the foundation for future studies in other globally significant bacterial pathogens when present as biofilms. Scientifically based strategies for management of pathogenic growth is necessary, particularly in this era of increasing antibiotic resistance.

**Graphic abstract:**

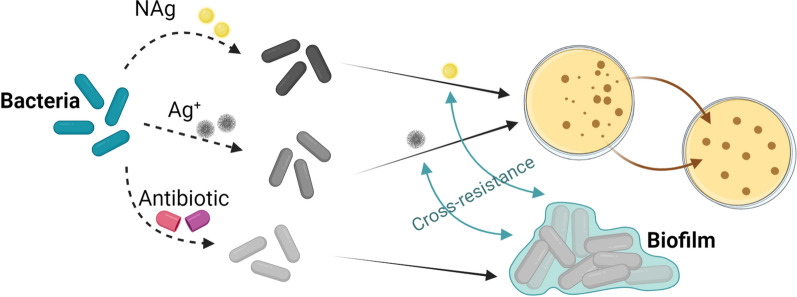

**Supplementary Information:**

The online version contains supplementary material available at 10.1186/s12951-021-01027-8.

## Background

**Fig. 1:**
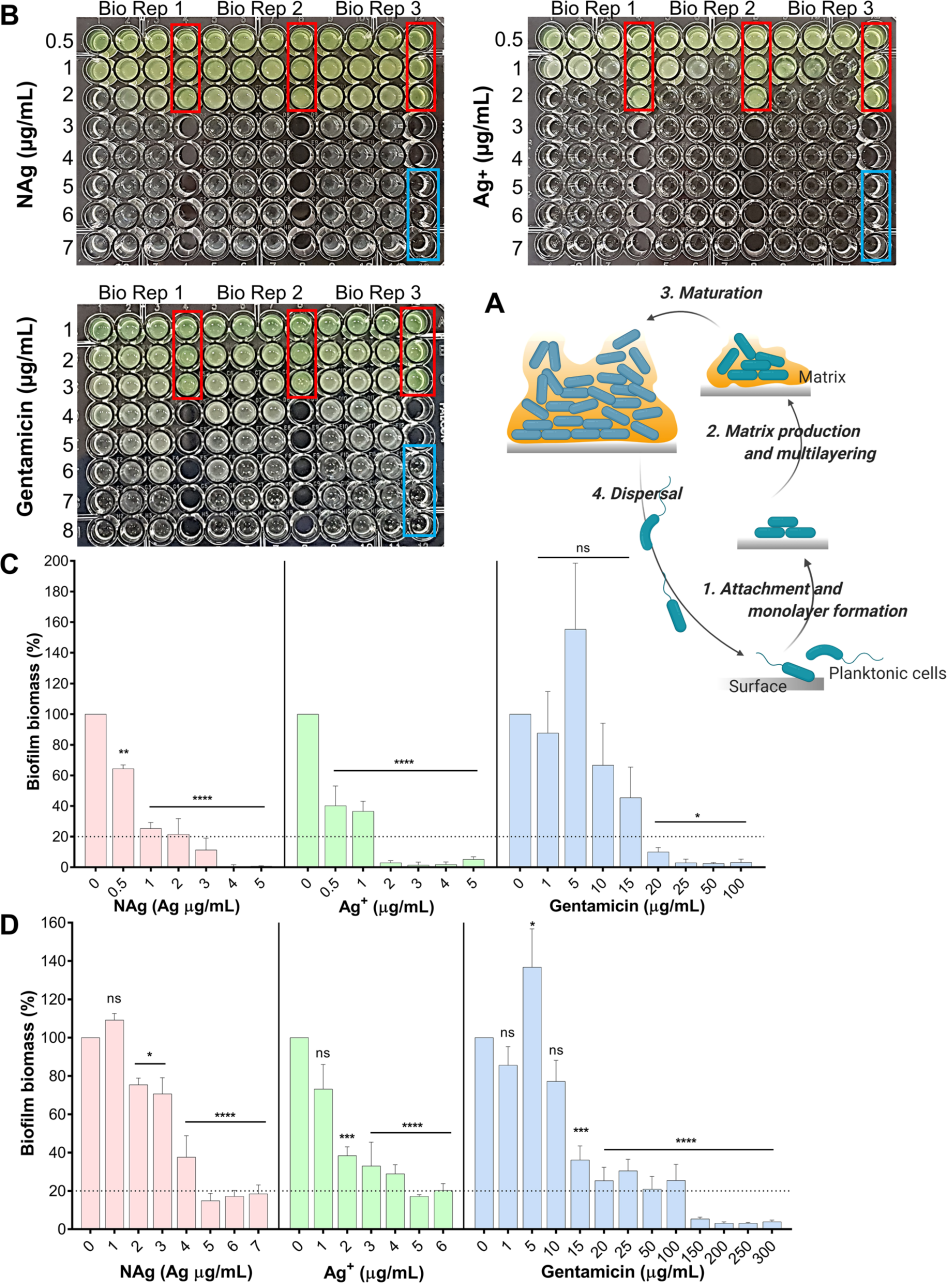
The inhibition of biofilm growth and eradication of established biofilm by NAg, ionic silver (Ag^+^) and gentamicin (GM). **A** A schematic of biofilm growth stages. **B** Determination of the minimum inhibitory concentration (MIC, defined as the lowest concentration that resulted in no visual growth) of NAg (tested at 0.5–7 µg/mL), Ag^+^ (0.5–7 µg/mL) and gentamicin (1–8 µg/mL) on *P. aeruginosa* general growth (24 h exposure at 37 °C). Red and blue boxes represent the untreated cell-only and the media-only controls, respectively. Effect of NAg, Ag^+^ and GM on **C** *P. aeruginosa* biofilm formation (24 h exposure, 37 °C), determining the minimum biofilm inhibitory concentration (MBIC, defined as the lowest concentration that caused ≥ 80% biofilm growth inhibition) and on **D** grown *P. aeruginosa* biofilm (24 h exposure, 37 °C), determining the minimum biofilm eradication concentration (MBEC, defined as the lowest concentration that caused ≥ 80% reduction of pre-formed biomass). Biofilm biomass is expressed as % relative to the cell-only control (no antimicrobial agent, 0 µg/mL). Error bars represent SEM (standard error of the mean) of three biological replicates (experiments with independent bacterial inocula from three isolates and different antimicrobial preparations, each with three technical replicates). * indicates statistically significant inhibition and eradication effects with p > 0.05 (not significant, ns), p < 0.05 (*), p < 0.01 (**), p < 0.001 (***) and p < 0.0001 (****), relative to the cell-only control

Biofilms, a surface-attached growth of microbes, are the dominant form of bacterial growth [1]. Bacterial biofilms are the leading cause of many chronic and persistent infections [2]. Their growth accounts for more than 50% of nosocomial (hospital-related) infections, being commonly found on medical devices and prostheses, such as intravenous and urinary catheters as well as cardiac pace makers, on wounds, even on heart valves [2, 3]. Treatment of biofilm-related infections is indeed a challenge. Unlike their planktonic counterparts, bacteria in biofilms are protected in a self-produced polymeric matrix, referred to as extracellular polymeric substance (EPS), rendering them resilient to antibiotics (Fig. 1A) [1]. This protective matrix acts as a barrier, significantly reducing the penetration of antibiotics, such that the concentration reaching the bacterial biomass is at sub-therapeutic levels and therefore ineffective [4, 5]. Bacteria in a clinically-relevant biofilm can generate signaling molecules (the auto-inducers) that activate the expression of a number of virulence genes, toxins as well as the EPS components, to safeguard the entire community from antibacterial agents [6, 7]. Bacteria in biofilms can also transfer antibiotic resistance genes to each other and at higher frequencies than their planktonic counterparts; with studies, for instance, reporting the transfer of ESBL genes-harbouring plasmids (the genes encode extended-spectrum β-lactamase enzymes that confer resistance to β-lactam antibiotics) in *Klebsiella pneumoniae* biofilm [8]. Collectively, these mechanisms help to protect biofilm cells from antibiotics and other stressors. This has prompted research on non-antibiotic therapy options for biofilm growth inhibition and eradication, with one of the major alternatives being the development of the broad spectrum antibacterial nanoparticles, nanosilver (NAg) [9].

NAg exhibit potent microbiological activities. The nanoparticle has shown efficacy on Gram-positive and Gram-negative bacteria, including those that cause nosocomial infections such as *Staphylococcus aureus* and *K. pneumoniae*, and indeed, at a growth inhibiting and even cell-killing concentrations that are non-toxic to human cells, as shown by many studies [10–13]. NAg, upon contact with an aqueous environment, will undergo oxidative dissolution and release silver ions (Ag^+^) [14, 15]. Studies have shown that both the leached soluble silver and the solid silver particulates that remain after leaching contribute to the overall toxicity of the nanoparticle [16, 17]. Unlike the single mode of action of most antibiotics, NAg attacks multiple targets in bacteria, from the cell envelope peptidoglycan and phospholipid molecules [18], the respiratory chain enzymes in the inner membrane, to disruption of iron-sulfur clusters that are present in many enzymes [19]. The latter is thought to lead to the release of the Fenton-active Fe^2+^ ions, which in turn reacts with the cellular peroxides to generate reactive oxygen species (ROS), one of the established mechanisms of NAg toxicity [18]. NAg is more widely used than the ionic form of silver, with the nanoparticle enabling a controlled release of Ag^+^, and therefore, a more sustained antibacterial activity [20]. The nanoparticles have also shown synergistic effects and enhance the antibacterial activity of conventional antibiotics, including on multidrug resistant bacteria [11, 21, 22].

There was a common perception that development of bacterial resistance to NAg is unlikely [14, 16, 18, 23], and yet, compelling scientific evidence in recent years has shown that bacteria can indeed adapt to the multi-targeting toxicity [23–26]. Gunawan et al. reported the growth of the environmental bacteria *Bacillus* sp. at the otherwise toxic NAg concentrations following prolonged exposures to the nanoparticle [24]. Such resistance development has also been found in other bacteria, including the clinically relevant *Escherichia coli* and *S. aureus* [23, 27], which is concerning as these ‘priority’ pathogens are known for their resistance to multiple antibiotics, including those used as the last line of treatment. Resistance to NAg has been associated with transient changes in cellular phenotypes (observable traits). For example, a strain of *E. coli* was found to increase its production of the primary flagella protein, flagellin, in the presence of the nanoparticle. This protein was shown to facilitate NAg aggregation and in turn, passivating the toxicity of the nanoparticle [25]. We have shown that *S. aureus*, can evolve gene mutations to develop stable resistance characteristics, which is maintained even after discontinuation of NAg exposure [23]. Resistance to NAg has also been linked to the presence of silver ion efflux systems, more specifically, the chromosomally encoded Omp/EnvZ porin and Cus efflux mechanisms, as well as the plasmid-encoded Sil efflux mechanisms [28]. Research inquiries into the nanoparticle resistance phenomena is still growing at this stage, with all studies, to the best of our knowledge, focused on the free-living planktonic bacterial growth. Bacteria, however, rarely exist as free-living entities and the possibility of NAg adaptation phenomena in the biofilm form of growth remains entirely unexplored. While quite a number of studies have shown the efficacy of NAg on bacterial biofilms [12, 13, 29–38], understanding the long-term impact of the nanoparticle exposures on the unique community growth is important [1, 3, 39]. The knowledge of adaptation phenomena in biofilms can inform strategies to control the most threatening form of pathogenic growth.

Herein, our study focused on *Pseudomonas aeruginosa* biofilms, by assessing the biofilm growth inhibition and eradication activity of NAg, in comparison to ionic silver and a model antibiotic. An estimated 1.7 million annual cases of nosocomial infections have been linked to biofilm growth, including those by *P. aeruginosa*, incurring a $94 billion healthcare cost per year, in the USA alone [40]. *P. aeruginosa* biofilms have been found growing on ventilators, causing pneumonia in patients, as well as in cystic fibrosis lungs, a major cause of mortality in CF patients [41, 42]. When growing as a biofilm, *P. aeruginosa* synthesizes three types of EPS polysaccharides; alginate, Psl and Pel as matrix barrier to protect the bacterial biomass from antibacterial agents [43–45]. *P. aeruginosa* is also known to take up resistance genes through cell-to-cell transfer of mobile genetic elements, with the phenomena being linked to the bacterium relatively high frequency of drug resistance [39, 46, 47]. Here, we explored the effect of long-term NAg exposures, in comparison to ionic silver and an antibiotic, assessing the potential development of adaptation mechanisms in *P.aeruginosa* biofilms. It is important to note that while there are some shared mechanisms, NAg in general exhibit different antibacterial activity when compared to ionic silver. For example, one study reported rapid stimulation of lethal cellular oxidative stress in *Bacillus subtilis* upon exposure to the nanoparticle, which is in contrast to the reactive oxygen species (ROS)-independent ionic silver toxicity mechanism on the bacterium [16]. Other study detected peptide dephosphorylation in NAg-treated *E. coli* and *Salmonella typhi* samples, while being absent in the ionic silver-treated samples [48]. The dephosphorylation is thought to inhibit cellular signalling in metabolic pathways, further linked to the observed growth inhibition. The present study found that, while the silver agents exhibit potent antibacterial effects on *P. aeruginosa* biofilm, the bacterium evolved persistence characteristics following prolonged exposures, highlighting the risks of long-term silver treatments.

## Results and discussion

### Biofilm inhibition and eradication activity of NAg, Ag^+^ and gentamicin

We first studied the toxic effects of NAg (finely dispersed *d*_TEM_ = ∼2 nm silver particulates on inert 30 nm TiO_2_ support) [14], Ag^+^ (supplied as AgNO_3_) and GM (gentamicin sulfate) on *P. aeruginosa*. The MIC, defined as the minimum concentration to inhibit bacterial growth, was observed at 3 and 2 µg/mL for NAg and Ag^+^, respectively (Fig. 1B). For *P. aeruginosa*, the NAg MIC has been reported to range between 0.59 to 50 µg/mL [12, 25, 26, 37, 49–52]. This is due to variations in the physico-chemical characteristics, such as size, shape, and presence of surface moieties, which significantly affect the antibacterial activity of the nanoparticle [53–55]. Our NAg MIC is in accordance with the lower range of the published MICs, which is most likely due to the relatively small particle size. Our Ag^+^ MIC is also consistent with those reported in the literature (0.002–1.88 µg/mL) for *P. aeruginosa* [26, 38, 56, 57]. Also seen in the present work, the generally greater antibacterial activity of Ag^+^ than NAg at equivalent silver concentration, which is thought to relate to the unique microbiological activities of the different silver species [16, 58]. Numerous studies have reported the higher extent of toxicity of Ag^+^ (free ions, when not locked in complexes) on bacteria than nanoparticulate silver [28, 59–61]. The MIC of GM was herein observed at 7 µg/mL, again, within the 2—32 μg/mL MIC range reported by earlier studies [52, 62–66]. Unlike the multi-targeting NAg and Ag^+^, GM typically works by binding to the 30S ribosomes in bacteria, in turn, inhibiting protein synthesis [67–69]. The antibacterial mechanisms of NAg and Ag^+^ are complex, which, among others, include cell envelope perforations, inhibition of enzymes and disruptions of structural proteins, as well as DNA damages [16, 18, 28, 59]. It is therefore reasonable to suggest that, relative to the antibiotic, it would be more challenging for bacteria to cope with NAg and Ag^+^ toxicity [70].

We studied the anti-biofilm effects of the antibacterial agents by determining the minimum concentrations to inhibit biofilm growth (MBIC), as well as, the minimum concentrations to eradicate an established biofilm (MBEC). Exposure of *P. aeruginosa* to increasing NAg and Ag^+^ concentrations saw a dose-dependent inhibition of biofilm growth, with the MBIC in this case defined as the minimum concentration that caused ≥ 80% growth inhibition relative to the cell-only control growth. The MBIC was observed at 3 µg/mL for NAg (Fig. 1C), which, as with the MIC, is consistent with the lower range published concentrations for *P. aeruginosa* (0.5 µg/mL to 1000 mg/mL) for smaller particle sizes [30, 33, 38, 49–51, 71, 72]. It is important to note that larger NAg particles have been reported to associate with higher MBICs [30, 38, 50, 71], indicating the significance of particle size in the anti-biofilm activity. The MBIC of Ag^+^ was 2 µg/mL (only limited number of Ag^+^ MBIC studies have been carried out at this stage for *P. aeruginosa*, including the ~ 10 µg/mL MBIC reported by Lemire et al.[73]). Our NAg and Ag^+^ MBICs are similar to their MICs and it is reasonable to suggest that these inhibitory concentrations should be within similar ranges, as biofilm formation would not occur if the general bacterial growth is inhibited [74, 75]. Earlier studies have linked the biofilm inhibition effect of silver to down-regulation of genes that are involved in biofilm formation as well as bacterial motility, and this is particularly evident with sub-inhibitory bacterial exposure to NAg [50]. With GM, a dose-dependent gradual decrease in biofilm growth was observed, with the MBIC determined at 15–20 µg/mL. Next, we studied the capability of these antibacterial agents to eradicate established biofilms. The MBEC is defined as the minimum concentration that caused ≥ 80% biomass reduction of a pre-formed biofilm relative to the cell-only control. As shown in Fig. 1D a dose-dependent biofilm eradication effect was seen for all agents, with an MBEC of 5 µg/mL for both NAg and Ag^+^. The NAg MBEC is consistent with the lower range of previously published concentrations (1.25 to 50 µ/mL) for smaller particle sizes [29, 30, 33, 35, 50]. The Ag^+^ MBEC is lower than the previously published values of 7 and 12.5 µg/mL (only limited number of Ag^+^ MBEC studies have been carried out for *P. aeruginosa*)[29, 73]. It has been hypothesized that Ag^+^ ions would pass through the surface porins that are present in biofilm cells [12, 38], while NAg, due to their solid particulate nature and larger size, may be less able to penetrate the biofilm matrix. In our study however, both silver were associated with similar MBECs. Although still unclear, the observations are in line with Park et al. reporting a comparable eradication activity of NAg and Ag^+^ on bacterial biofilms under stirring conditions, which was indicated to enhance the biosorption of NAg into the biomass, rendering the nanoparticle as effective as the ion [76]. For GM, although a reduction in biofilm biomass was observed at concentrations starting from 10 µg/mL, the ≥ 80% biomass reduction threshold was only seen at the 100–150 µg/mL range, considered herein as the MBEC. The GM MBEC for *P. aeruginosa* has been previously reported at 16 µg/mL [77], and the difference could be related to the different strains used (ATCC 27,853 in their study). Overall, our MIC, MBIC and MBEC data has shown that NAg, Ag^+^ and GM are capable of both inhibiting biofilm formation as well as eradicating established biofilms of *P. aeruginosa*. However, as next described, the bacterium is developing adaptation mechanisms upon prolonged exposures and that these mechanisms are unique for the silver and antibiotic.

### Adaptation responses of biofilm-forming *P. aeruginosa* to NAg, Ag^+^ and gentamicin

**Fig. 2:**
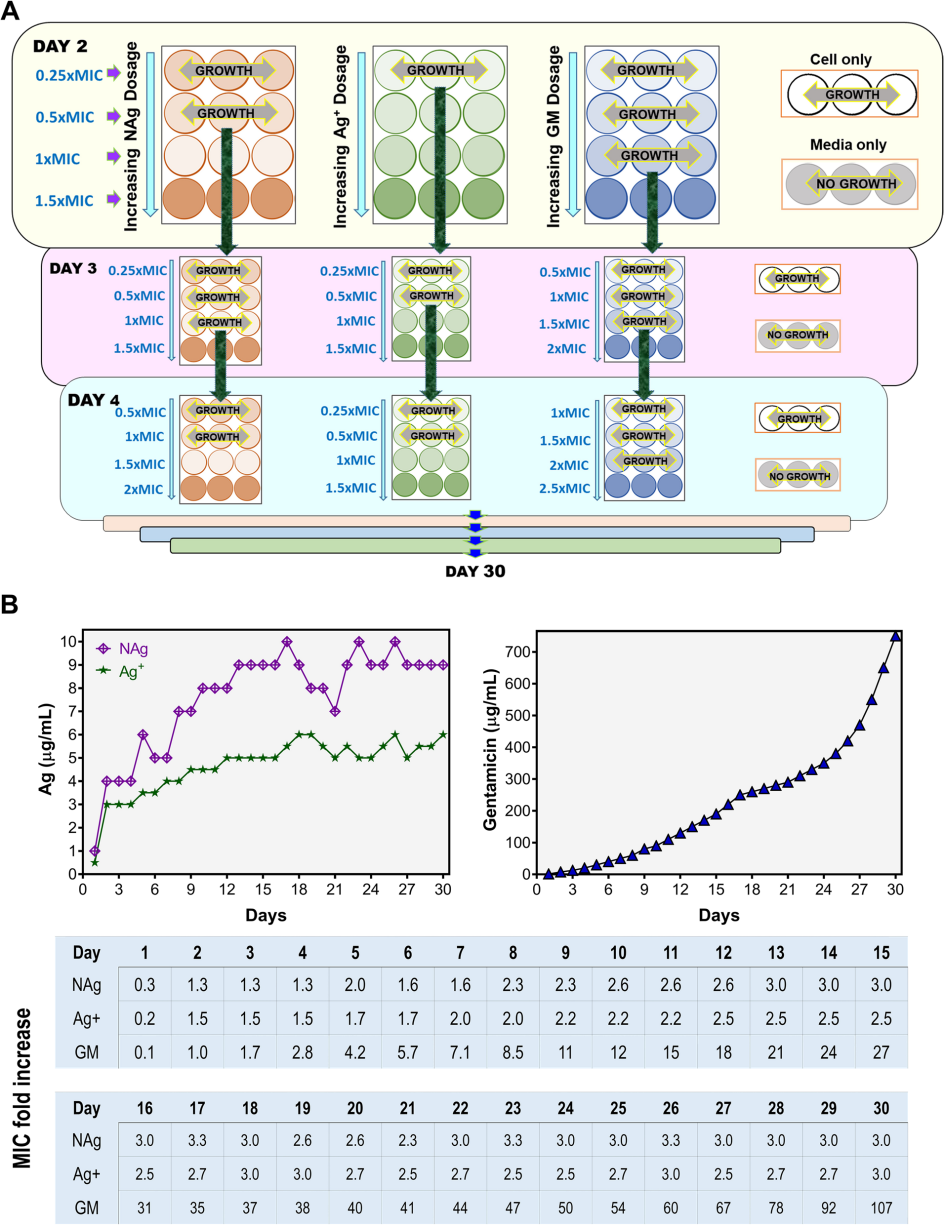
Evolutionary adaptation of *P. aeruginosa* to NAg, ionic silver (Ag^+^) and gentamicin (GM) through serial passaging. **A** A schematic of the passaging experiment. Starting at the respective sub-MIC levels of each agent, the biofilm-forming bacterium was continuously exposed to progressively increasing concentrations of NAg, Ag^+^ and GM for 30 d via sub-culturing every 24 h. The passaging experiment also included a cell-only passaged culture (no antibacterial agent). **B** The increasing shifts in the highest dosage of NAg, Ag^+^ and GM at which the bacterium could proliferate, with the table detailing the shifts as MIC fold-increase for each agent. Two biological replicates were performed for the passaging experiments, each with three technical replicates. Refer to Additional file 1: Figure S1 for the second biological replicate data

The ability of *P. aeruginosa* to adapt and evolve resistance was studied with a 30 d exposure experiment by serially passaging (sub-culturing every 24 h) the bacterium in increasing concentrations of NAg, Ag^+^ and GM (Fig. 2A). The prolonged exposure saw progressive shifts in the highest antimicrobial dosage at which *P. aeruginosa* could proliferate (Fig. 2B). For NAg, starting at sub-MIC dosage (1 µg/mL) at day 1, the bacterium could grow at increasing nanoparticle dosages, reaching a maximum at 9 µg/mL (threefold MIC) at day 30. Similarly, for Ag^+^, *P. aeruginosa* was able to proliferate when subjected to increasing silver dosage from sub-MIC (0.50 µg/mL) to 6 µg/mL (threefold MIC) at day 30. By comparison, the exposure to GM resulted in a drastic increase, starting at sub-MIC dosage and reaching 750 µg/mL (100-fold MIC) at day 30.

**Fig. 3:**
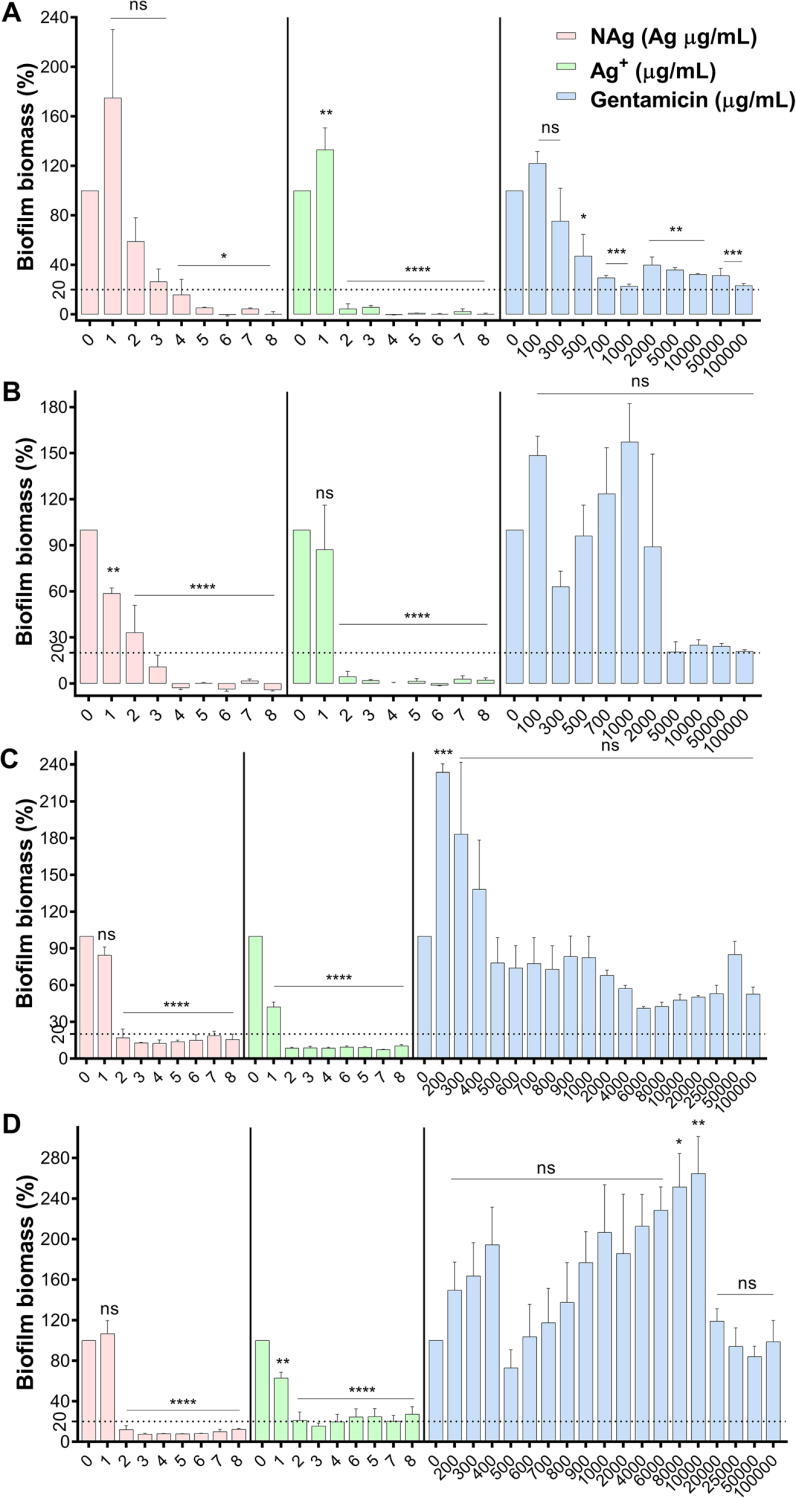
Post long-term exposure changes in the MBIC **A**, **B** and MBEC **C**, **D** of NAg, Ag^+^ and gentamicin (GM) on the respective mid-point passaged **A**, **C** and end-point passaged **B**, **D** strains (24 h exposure, 37 °C). Biofilm biomass is expressed as % relative to the cell-only control (no antimicrobial agent, 0 µg/mL). Error bars represent SEM (standard error of the mean) of three biological replicates (experiments with independent bacterial inocula from three isolates and different antimicrobial preparations, each with three technical replicates). *Indicates statistically significant biofilm growth inhibition **A**, **B** and eradication of established biomass **C**, **D** with p > 0.05 (not significant, ns), p < 0.05 (*), p < 0.01 (**), p < 0.001 (***) and p < 0.0001 (****), relative to the cell-only control. The MBIC and MBEC studies of the second biological replicate of the passaging experiment are presented in Additional file 1: Figure S2

The observed growth at increasing concentrations of NAg, Ag^+^ and GM during the passaging experiment indicated that the bacterium has developed adaptation responses. Bacteria have been known to adapt to elevated levels of antimicrobials via a number of different mechanisms. A resistance trait is developed when a higher dosage of the antibacterial agent is required to inhibit the growth, characterized by an MIC increase, while a tolerance or persistence manifest when it takes longer for the agent to kill the bacterial population, with no change in the MIC [78]. To first determine if *P. aeruginosa* has developed resistance, the passaged cultures were sampled from the mid-point of the passaging course (at between 12 and 17 d), as well as at the end of the experiment, and were re-exposed to the respective antimicrobials, to look for a minimum two-fold increase in the MIC, MBIC and MBEC. For the general growth, there was no increase in the MIC of both NAg (3 µg/mL) and Ag^+^ (2 µg/mL) for the mid-point as well as end-point passaged cultures relative to the wild-type *P. aeruginosa*. For GM, a 40 to 60-fold MIC increase (from 7 to 300–400 µg/mL) and 200 to 300-fold (from 7 to 1500–2000 µg/mL) were seen for the mid-passaged and end-passaged cultures, respectively. Similarly, biofilm inhibition studies revealed comparable MBIC of NAg and Ag^+^ for the mid-passaged (NAg at 4 µg/mL and Ag^+^ at 2 µg/mL) and end-passaged (NAg at 3 µg/mL and Ag^+^ at 2 µg/mL), as those of the wild-type strain. In contrast, for GM, the ≥ 80% growth inhibition threshold shifted to 1000 µg/mL for both the mid-passaged and end-passaged cultures (Fig. 3A, B) (note that in other independent replicates of GM-passaged cultures, the inhibition threshold were only observed at the highest test-able concentration of 100 mg/mL, reaching the solubility limit of the antibiotic, Additional file 1: Figures S1 and S2). For the biofilm eradication, the MBEC of NAg for the mid- and end-passaged cultures were 2 µg/mL, even lower than that of the wild-type strain at 5 µg/mL (Fig. 3C, D). The MBEC of Ag^+^ for both passaged cultures (2 µg/mL) were also lower than the wild-type culture. For GM, essentially no biofilm eradication was observed even at 100 mg/mL, for both the mid- and end-passaged cultures.

Taken together, our MIC, MBIC and MBEC assessments of the antibacterials on the passaged cultures indicate that *P. aeruginosa* did not develop resistance to the silver agents, with still similar minimum concentrations required to inhibit the biofilm formation or to eradicate grown biofilm, as the wild-type strain. Instead, the bacterium was found to exhibit other types of adaptation as a result of the prolonged exposures, which will be described in the next section. In the case of NAg, the findings are in contrast to the only earlier evidence of potential resistance development in *P. aeruginosa*. Panacek et al. reported a 32-fold MIC increase following a 20 d exposure to increasing NAg concentrations [25]. The discrepancies with the present work could result, at least in part, from the different approach of the prolonged exposures, whereby a 2-to-3 d cell culturing in medium only (no NAg) was included by Panacek et al. in between each 24 h passaging sequence, while herein, the bacterium was constantly exposed to NAg. To the best of our knowledge, the previous work did not explore the potential for adaptation responses in the biofilm form of *P. aeruginosa* growth. In the case of Ag^+^, Muller and Merrett reported that *P. aeruginosa* is intrinsically resilient to the ion with an MIC of 20 µg/mL, which is thought to associate with the bacterium’s ability to produce pyocyanin, a redox molecule that can reduce Ag^+^ to Ag^0^ particulates, thereby lowering the toxicity [79]. The work however, did not investigate the potential of the bacterium to develop adaptation during prolonged exposure. Here, the GM-passaged *P. aeruginosa* developed resistance with significant increase in MIC, MBIC and MBEC, and consistent with earlier reports, the resistance traits developed rapidly [80, 81]. Resistance to GM and other aminoglycosides typically result from the activity of specific aminoglycoside-modifying enzymes, which can lead to the inactivation of aminoglycosides, rendering them unable to recognise their 30S ribosomal target in bacteria [82]. Other studies have linked the resistance mechanism to a reduced cell envelope permeability, while some have suggested the role of an efflux mechanism (the MexXY system) [82–84]. The development of the GM-resistant culture in the present work verifies our sequential passaging methodology for the evolutionary study of adaptation responses.

### Persistence mechanism to NAg and Ag^+^

Since it was clear that the silver exposure did not result in the development of resistance, we next tested whether the passaged strains were demonstrating tolerance or persistence. More specifically, tolerance is the ability of bacterial population in general to survive longer in the presence of antimicrobials as a result of a slower killing rate, whereas, persistence has the additional attribute of affecting only a subpopulation of cells, the so-called ‘persisters’, to survive exposure to an otherwise lethal antibacterial concentration. This slower killing trait is characterized by an increase in the minimum exposure time required to kill 99% and 99.99% of the population, referred to as MDK_99_ and MDK_99.99_, for tolerance and persistence, respectively [78]. Time-kill studies were performed to determine any increase in these parameters for NAg and Ag^+^ for the passaged cultures relative to the wild-type strain.

**Fig. 4:**
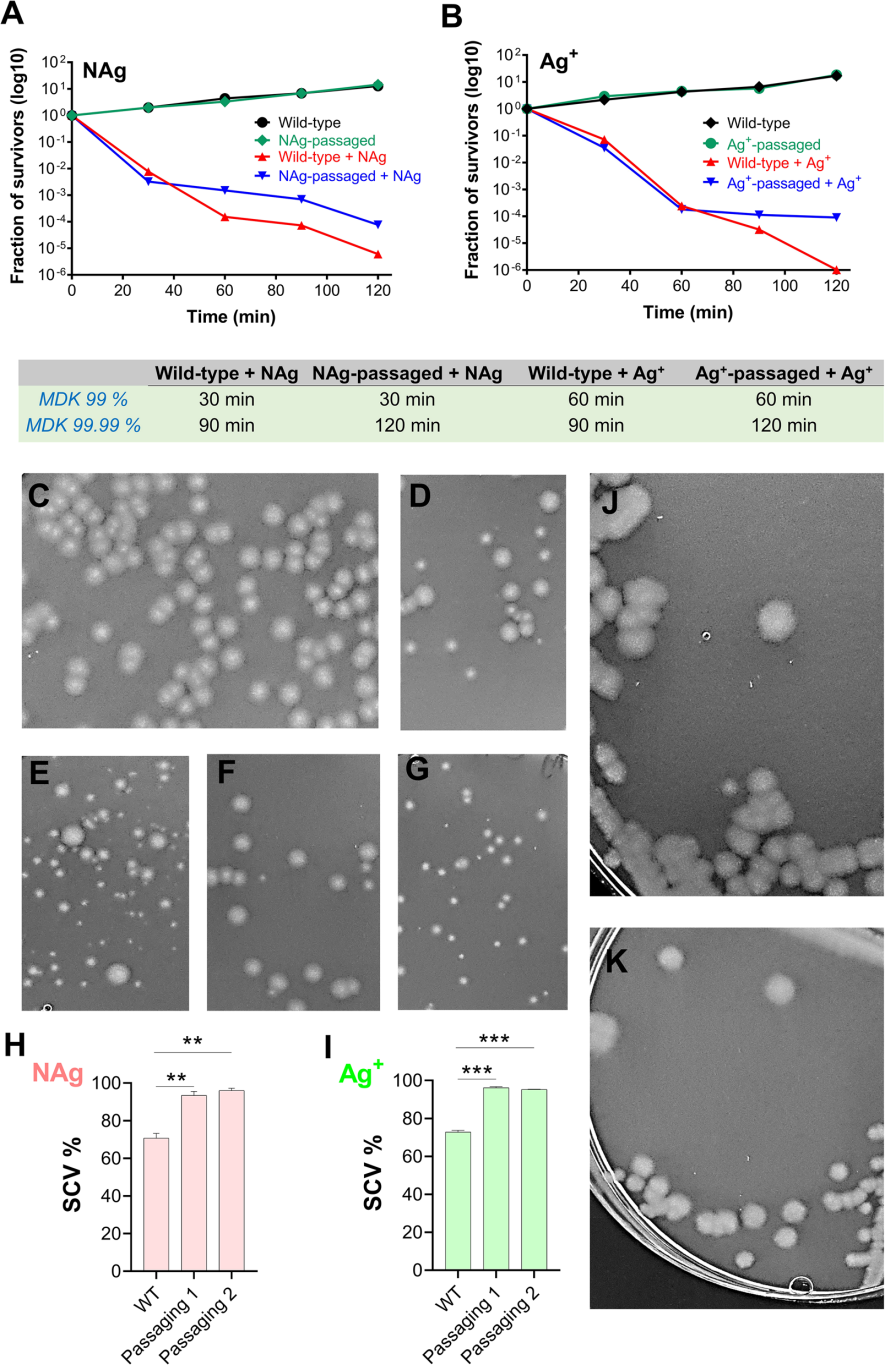
Persistence to silver and the formation of small colony variants (SCVs) in *P. aeruginosa*. Killing kinetics of **A** NAg-passaged and **B** Ag^+^-passaged *P. aeruginosa* in comparison to the wild-type strain. The bacterium was first grown to early exponential phase and exposed to the silver antibacterials at their respective 1.5 × MIC dosages (4.5 µg/mL for NAg, 3 µg/mL for Ag^+^). The log_10_ decrease in cell population was determined relative to population at time 0 (as colony forming units). Refer to Additional file 1: Figure S3A, B for the biological replicate of the killing kinetics and to Additional file 1: Figure S3C, D for the two biological replicates of the killing kinetics of the strains isolated from the second biological replicate of the NAg and Ag^+^ passaging experiments. **C** Representative image of normal colonies of non-silver-treated wild-type, NAg-passaged and Ag^+^-passaged strains, **D**, **E** The colonies from the wild-type and NAg-passaged strains after 60 min exposure to NAg (at 1.5 × MIC dosage), **F**, **G** The colonies from the wild-type and Ag^+^-passaged strains after 60 min exposure to Ag^+^ (at 1.5 × MIC dosage). Note the occurrence of SCVs with the silver treatments. **H**, **I** The SCVs quantification in the silver-treated samples is presented as % relative to the total number of colonies and shown is the data from the two biological replicate of the NAg and Ag^+^ passaging experiments. *Indicates statistically significant higher occurrence of SCVs with p < 0.01 (**) and p < 0.001 (***), relative to the wild-type strain. The growth of normal colonies resumed upon sub-culturing of the SCVs from the (J) NAg-passaged and (K) Ag^+^-passaged strains, on antimicrobial-free medium

Exposure of the NAg- and Ag^+^-passaged cultures to the respective silver species (at 1.5 × MIC dosages) resulted in rapid killing of both bacterial populations in the first 30 min. The rapid killing was followed by a slower killing of the passaged culture cells when compared to the wild-type. This biphasic cell-killing profile is consistent with persistence (Fig. 4A, B) [78]. The killing kinetics of the NAg-passaged culture saw a longer MDK_99.99_ relative to the wild-type, with no change in MDK_99_, indicating the development of persistence in the passaged culture (Fig. 4A). Similarly, a longer MDK_99.99_ and no change in MDK_99_ was also seen for Ag^+^ (Fig. 4B). Persistence develops when bacteria undergo non-mutational variations such that a sub-population of cells are capable of surviving bactericidal concentrations of an agent for longer than the main population [78, 85–87]. These dormant, metabolically inactive cells are not considered ‘resistant’, as resistance is the ability to both survive and proliferate at an otherwise toxic concentration of an agent [78, 85]. Many studies have linked persistence to the formation of small colony variants (SCVs) in a bacterial population [88, 89]. Our work have shown that prolonged exposures of *P. aeruginosa* to NAg and Ag^+^ had indeed resulted in the formation of persister cells in the form of SCVs (Fig. 4D–G). While the variants were found in the wild-type strain, their abundance were ~ 25% higher in both the NAg- and Ag^+^-passaged strains (Fig. 4H, I). Further, in addition to their smaller colony size, SCVs often show increased exopolysaccharide production and enhanced biofilm formation [90]. The latter was also indicated in the present study, with both the NAg- and Ag^+^-passaged strains forming an at least two-fold higher biofilm biomass than the wild-type strain (Additional file 1: Figure S3).

To the best of our knowledge, this is the first evidence for the development of persistence phenotype in bacteria in response to silver antibacterials. Studies have shown that oxidative stress, one of the major toxicity mechanisms for silver, can trigger a persistence phenotype and formation of SCVs [91–94]. Further, persister cells have been associated with an increased expression of efflux pumps [93], the latter were already seen in bacteria with an increased resilience to silver (Ag^+^) [91]. A link has also been established between the formation of SCVs to the increased expression of the secondary messenger molecule cyclic-di-GMP in *P. aeruginosa*, which regulates many processes involved in virulence as well as the transition between planktonic to biofilm growth [95]. Finally, studies have shown that persister cells can resume growth once the antibacterial exposure ceases [87, 96, 97]. Indeed, we observed normal colony growth following sub-culturing of the SCVs, from both the NAg- and Ag^+^-passaged cultures, on antimicrobial-free medium (Fig. 4J, K). Thus, it is apparent that silver exposure selects for persister cells in the population as well as SCVs, which is linked to survival of *P. aeruginosa* in the presence of the antibacterials.

### Biofilm-associated silver and antibiotic cross-resistance

**Fig. 5:**
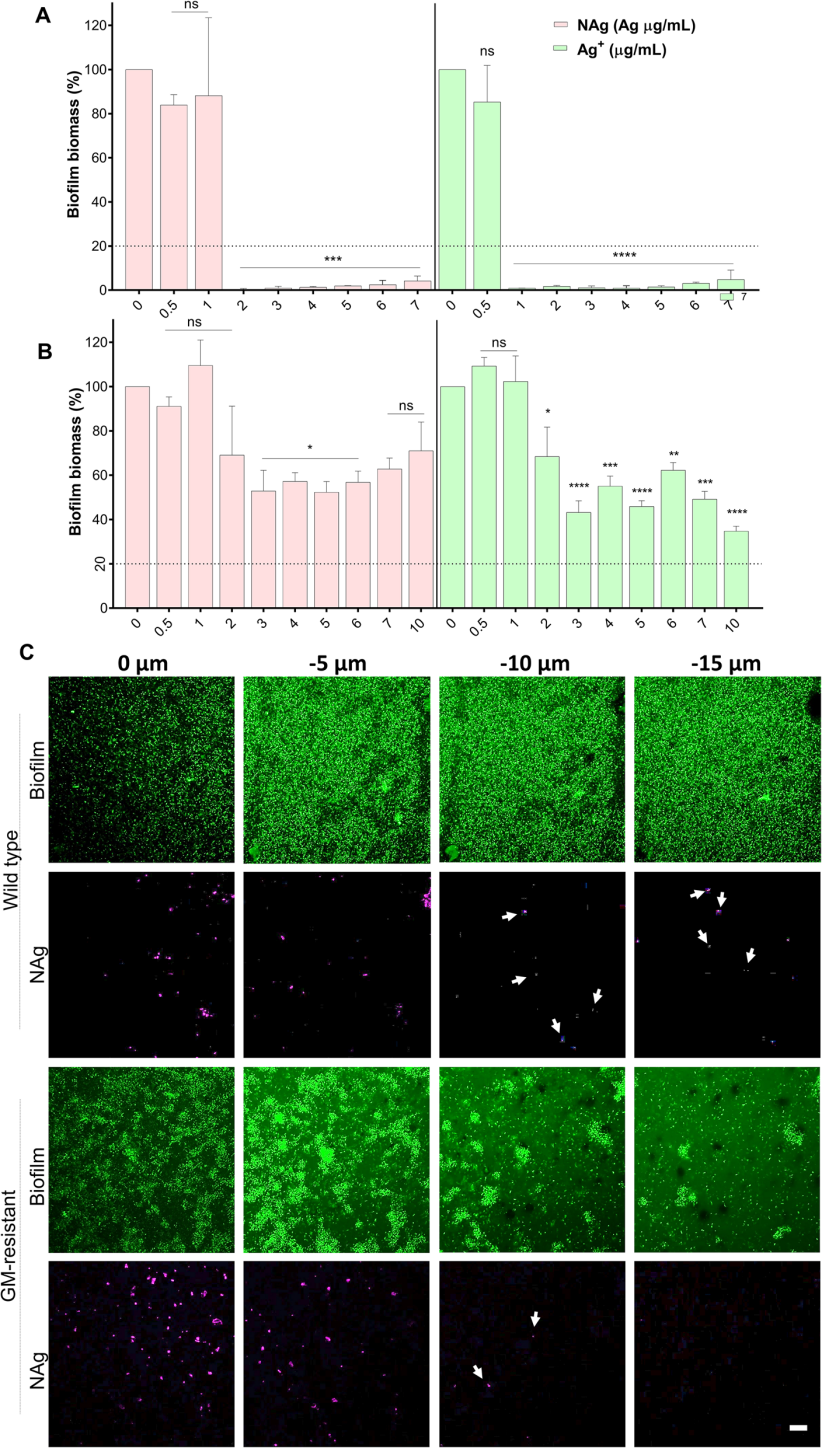
Biofilm-associated cross-resistance of gentamicin (GM)-resistant strain to silver. **A** Inhibition of biofilm growth of *P. aeruginosa* GM-resistant strain by NAg and Ag^+^ (24 h exposure, 37 °C). **B** Eradication of established biofilm of GM-resistant strain by NAg and Ag^+^ (24 h exposure, 37 °C). Biofilm biomass is expressed as % relative to the cell-only control (no antimicrobial agent, 0 µg/mL). Error bars represent SEM (standard error of the mean) of three biological replicates (experiments with independent bacterial inocula from three isolates and different antimicrobial preparations, each with three technical replicates). *Indicates statistically significant inhibition and eradication effects with p > 0.05 (not significant, ns), p < 0.05 (*), p < 0.01 (**), p < 0.001 (***) and p < 0.0001 (****), relative to the cell-only control. **C** Penetration of NAg particle (aggregates) in wild-type and GM-resistant *P. aeruginosa* biofilms. Biofilm biomass (green) were exposed to NAg at 20 × MIC dosage (3 h, 37 °C) and sectional z-stack depth images were acquired using multiphoton microscope. The second harmonic generation and hyper Rayleigh scattering signals of NAg particles (pink) were used to detect their presence in the biofilms. Scale bar = 20 µm. Refer to Additional file 1: Figure S4 for the NAg and Ag^+^ biofilm inhibition and eradication studies, as well as the multiphoton biofilm-nanoparticle imaging of the GM-resistant strain obtained from the second biological replicate of the passaging experiment

Finally, we studied the evolved GM resistance for the possibility of cross-resistance to the silver antibacterials. The MIC and MBIC of NAg and Ag^+^ on the GM-resistant *P. aeruginosa* was found at 2 and 1 µg/mL, respectively (Fig. 5A), and these were lower than those observed for the wild-type strain (NAg at 3 µg/mL and Ag^+^ at 2 µg/mL). The data show that the GM-resistant strain is more sensitive to both NAg and Ag^+^. This could relate to the known modification of the 30S ribosomal subunit linked to the GM resistance [98]. However, how that would lead to the enhanced silver sensitivity is not currently clear. Further silver studies on an already grown GM-resistant *P. aeruginosa* biofilm yields an interesting contrast. As shown in, the MBEC were considerably higher with no significant biofilm eradication observed even at 10 µg/mL, for both silver formulations (Fig. 5B). Note that the MBEC was at 5 µg/mL NAg and Ag^+^ for the wild-type biofilm. The findings indicate cross-resistance traits to NAg and Ag^+^ of the GM-resistant strain when the bacteria have already formed a biofilm, but apparently not prior, when the population were still in their free-living planktonic form. The cross-resistance phenomena were most likely associated with a hindered biofilm penetration of the silver antibacterials. Using multiphoton microscopy, we detected presence of NAg at up to 10 µm depth in the GM-resistant biofilm samples, while the nanoparticles were still present at up to 15 µm depth in the wild-type biofilm (Fig. 5C*,* Additional file 1: Figure S4). Although the exact mechanism remains to be explored, the hindered penetration is thought to result, at least in part, from changes in the biofilm EPS matrix composition. We observed the formation of cell aggregates in the GM-resistant biofilm, while these ‘clumps’ of cells were absent in the wild-type biofilm (Fig. 5C). Earlier studies have noted similar phenotype in a *P. aeruginosa* strain that overproduces one of the major biofilm polysaccharide matrix components, the Psl, in turn, enhancing cell-to-cell adhesion [99].

## Conclusions

In summary, the work reported unique silver adaptation mechanisms with pathogenic biofilm-forming bacterium that have not been previously reported with planktonic bacterium studies. While silver nanoparticles (NAg) and ionic silver (Ag^+^) are effective in inhibiting and eradicating *P. aeruginosa* biofilm growth, we found evidence of persister cells as a mechanism of survival upon repeated NAg and Ag^+^ exposures. Our work highlights for the first time the possibility of recurrent infections with prolonged silver treatments, with persister cells and the associated small colony variant (SCV) phenotype. As expected, the biofilm-forming bacterium rapidly developed a high level of resistance to the antibiotic gentamicin (GM). We further found that the antibiotic-resistant strain was in fact sensitive to NAg and Ag^+^ when in the planktonic phase, with both silver formulations capable of inhibiting the biofilm growth, however, not when the resistant strain was already forming biofilm. Both NAg and Ag^+^ were ineffective in eradicating grown GM-resistant biofilm, with the apparent silver/antibiotic cross-resistance being linked to the hindered penetration of the silver nanoparticles. Taken together, the findings inform strategies on the control of pathogenic biofilm growth. NAg (and Ag^+^) are not recommended for prolonged treatments of biofilm infection, whereas both silver formulations are effective in inhibiting antibiotic-resistant biofilm growth, although not on established biomass. Future studies should explore the NAg adaptation mechanisms of other important pathogenic species in their biofilm form of growth, as well as the potential for the nanoparticle as an alternative to combat the antibiotic-resistant growth.

## Materials and methods

### Bacterial strain and preparation of bacterial inoculum and antibacterial agents

*Pseudomonas aeruginosa* PAO1, a derivative of the PAO strain first sourced from the wound of an Australian burn victim, was used in this study due to its innate ability to form biofilms [100]. Cultures were grown in cation adjusted Mueller–Hinton agar or broth (CAMH, BD, Australia). To prepare the bacterial inoculum for each experiment, an overnight culture was grown at 37 °C, 250 rpm orbital shaking for 16–17 h, then pre-conditioned by 1/100 dilution with fresh CAMHB and incubated for a further 2 h at 37 °C, 250 rpm to reach ~ 10^7^ cfu/mL bacterial concentration. This process rendered the culture to enter the log-phase, whereby the cells are in their most metabolically active state, to test the effects of antibacterial agents [101].

Nanosilver (NAg) with Ag_2_O finely dispersed onto inert TiO_2_ support was prepared by flame spray pyrolysis at the University of New South Wales [24]. For subsequent use in antibacterial studies, NAg was sterilized by gamma-irradiation with Co-60 source at 6.7 Gy/min for 1 h (mean = 402 Gy/h) at the Australian Nuclear Science and Technology Organization. Silver nitrate (AgNO_3_, Merck & Co., Inc., USA) dissolved in Milli-Q water served as ionic silver (Ag^+^) source and gentamicin (GM) was supplied as gentamicin sulfate (Sigma-Aldrich, USA) dissolved in Milli-Q water. Immediately before the experiments, NAg suspension was homogenised by ultrasonication (Vibra-cell, Sonics & Materials, Inc.) at 50% output control for 20 s. Experiments with NAg and AgNO_3_ were performed under dark conditions to render the TiO_2_ support of the nanoparticles photocatalytically inactive and to prevent Ag^+^ reduction to Ag^0^, respectively.

### Determination of minimum inhibitory concentration (MIC), minimum biofilm inhibitory (MBIC) and eradication concentration (MBEC)

MIC was determined following the broth microdilution method by the Clinical and Laboratory Standard Institute with modifications. Briefly, the bacterial inoculum was exposed to various concentrations of the antibacterial agents in CaMH broth in a 96-well microtiter plate (Corning, USA) at 160 µL total volume, with a cell-only and broth-only controls. Plates were sealed with AeraSeal (Excel Scientific, USA) and incubated in a static humidified incubator at 37 °C for 24 h. The MIC was determined visually as the lowest antibacterial concentration with no observable growth. The experiments were performed in three biological replicates, each with three technical replicates.

For MBIC studies, similar exposure systems were set up. After the 24 h exposure in a static humidified incubator at 37 °C, the culture wells were washed three times with phosphate buffered saline (PBS) using an auto-plate washer (Bio-Tek, ELx405, USA) to remove any planktonic cells, followed by staining of the biofilm biomass with 0.2% (v/v) crystal violet (CV) at room temperature for 45 min on a plate shaker at ~ 75 rpm (Bio-line, Edwards Instrument Company, AUS). The biomass was then washed three times with PBS, followed by a de-staining step with 33% (v/v) acetic acid for 20 min at room temperature at ~ 75 rpm to release any biofilm-bound CV into the solution. The absorbance was measured at 595 nm in a microplate reader (Tecan Infinite M200 Pro). The MBIC was herein defined as the lowest antibacterial concentration that caused ≥ 80% growth inhibition relative to the cell-only control.

For MBEC studies, biofilms were first grown in a 96-well plate from 80 μL bacterial inoculum and incubated in a humidified incubator at 37 °C for 24 h. The culture wells were then washed three times with PBS to remove any planktonic cells. The pre-formed biofilms were exposed to various concentrations of the antibacterial agents (160 µL total volume) for 24 h at 37 °C. The biofilm biomass was stained with CV and destained with acetic acid as previously described. The MBEC was herein defined as the lowest antibacterial concentration caused ≥ 80% biomass reduction of a pre-formed biofilm relative to the cell-only control.

### Evolutionary adaptation to NAg, Ag^+^ and gentamicin

To investigate the potential for *P. aeruginosa* to evolve adaptation to NAg, Ag^+^ and GM, the bacterium was serially passaged (via sub-culturing every 24 h) for 30 d in 24-well microtiter plates (Corning, USA) in the presence of progressively increasing concentrations of the antibacterial agents in CaMH broth, starting at their respective sub-MIC doses. At day 1 for example, the bacterial inoculum at 1/10 dilution was exposed to 0.25 × , 0.5 × , 1 × and 1.5 × MIC of the agents at 1 mL total volume, with a cell-only and broth-only controls (Fig. 2A). Three technical replicates were set up for each of the antibacterial dosages. Following 24 h exposure at 37 °C, 150 rpm, cultures from the highest antibacterial exposure concentration that allowed visible growth were passaged at 1/10 dilution to the next higher antibacterial dosage range, for instance, at 0.5 × , 1 × , 1.5 × and 2 × MIC. The passaging experiments were carried out with two independent biological replicates. The MIC, MBIC and MBEC of NAg, Ag^+^ and GM on the mid-point (between 12 to 17 d) and end-point (30 d) passaged strains were determined as previously described, to confirm for resistance development (a minimum of two-fold increase relative to the wild-type strain). The antibiotic GM is to also serve as a reference for validation of the passaging experiment as *P. aeruginosa* has been known to develop resistance to GM more rapidly when compared to many antibiotics [80, 81].

### Time-kill assay

Early-exponential phase cultures of the NAg- and Ag^+^-passaged strains were exposed to the respective silver antibacterials at their 1.5 × MIC dosages at 37 °C, with samples taken every 30 min for 2 h. The samples were serially diluted and plated on CaMH agar and incubated for 24 h at 37 °C for growth of colonies. The time-kill profiles were obtained by plotting the fraction of surviving cells at each time point (as cfu (colony forming units)/mL) relative to the time zero cell population. Two independent time-kill biological replicates were carried out for each of the two biological replicates of the NAg- and Ag^+^-passaged strains.

### Fluorescence imaging of biofilms and NAg penetration

To establish the biofilms, a measured 200 µL of the bacterial inoculum was inoculated to a glass-bottomed Ibidi imaging well slide (DKSH, Aus) and incubated at 37 °C for 18 h. NAg particles were then dosed to the biofilms at × 20 MIC dosage for 3 h. After exposure, the unabsorbed/unadsorbed particles were removed by three times PBS washing over the surface of the biomass. The biomass was stained with rhodamine b (200 µL, 5 µg/mL) for 5 min at 37 °C. Excess stain was removed and replenished with CaMH broth for imaging. Imaging was performed with a multiphoton microscope LSM 710 with Axio Observer Z1 (Carl Zeiss, Jena, Germany) equipped with a tunable titanium sapphire laser Mai Tai® (Spectra Physics, Mountain View, CA, used at 6 mW laser power). Images were taken in the optical z-direction with 5 µm steps. The rhodamine b that stained the biomass was imaged at ex:800 nm two photon em:508–710 nm and the second harmonic generation and hyper Rayleigh Scattering signals of the NAg were imaged simultaneously in a separate channel at ex:800 em:370–420 nm and 26% laser power [102, 103]. All samples were imaged in triplicates and representative samples are shown.

### Statistical analysis

Statistical analysis was performed using GraphPad Prism and the results were compared using a one-way analysis of variance (ANOVA), followed by Dunnett’s multiple comparison test, with a p value significance level set at < 0.05.

## Supplementary Information

**Additional file 1: Figure S1.** Biological replicate of *P. aeruginosa* serial passaging in the presence of progressively increasing concentrations of NAg, Ag^+^ and GM. **Figure S2.** Post long-term exposure changes in the MBIC and MBEC of NAg, Ag^+^ and GM on the respective mid-point passaged and end-point passaged cultures from the second biological replicate of the passaging experiments. **Figure S3.** **A**–**D** Killing kinetics of NAg-passaged and Ag^+^-passaged *P. aeruginosa* in comparison to the wild-type strain. **E** Biomass quantification of biofilms grown from NAg-passaged and Ag^+^-passaged strains in comparison to the wild-type strain. **Figure S4.** **A**, **B** Inhibition and eradication of biofilm growth of *P. aeruginosa* GM-resistant strain (obtained from the second biological replicate of the passaging experiments) by NAg and Ag^+^. **C** Penetration of NAg particle (aggregates) in wild-type and GM-resistant *P. aeruginosa* (obtained from the second biological replicate of the passaging experiments).

## Data Availability

The datasets generated and analysed during the current study are available from the corresponding author on reasonable request.
