# Supplementary material for: Evolution of biofilm-forming pathogenic bacteria in the presence of nanoparticles and antibiotic: adaptation phenomena and cross-resistance
Source: J Nanobiotechnology. 2021 Sep 27;19:291. doi: 10.1186/s12951-021-01027-8 (PMC8474960; doi:10.1186/s12951-021-01027-8)
Supplement: Supplementary file 1 — Additional file 1: Figure S1. Biological replicate of P. aeruginosa serial passaging in the presence of progressively increasing concentrations of NAg, Ag+ and GM. Figure S2. Post long-term exposure changes in the MBIC and MBEC of NAg, Ag+ and GM on the respective mid-point passaged and end-point passaged cultures from the second biological replicate of the passaging experiments. Figure S3. A–D Killing kinetics of NAg-passaged and Ag+-passaged P. aeruginosa in comparison to the wild-type strain. E Biomass quantification of biofilms grown from NAg-passaged and Ag+-passaged strains in comparison to the wild-type strain. Figure S4. A, B Inhibition and eradication of biofilm growth of P. aeruginosa GM-resistant strain (obtained from the second biological replicate of the passaging experiments) by NAg and Ag+. C Penetration of NAg particle (aggregates) in wild-type and GM-resistant P. aeruginosa (obtained from the second biological replicate of the passaging experiments). [file 12951_2021_1027_MOESM1_ESM.docx]

**Additional file**

Evolution of Biofilm-Forming Pathogenic Bacteria in the Presence of Nanoparticles and Antibiotic: Adaptation Phenomena and Cross-Resistance

*Riti Mann,^1^ Amy Holmes,^2^ Oliver McNeilly,^1^ Rosalia Cavaliere,^1^ Georgios A. Sotiriou,^3^ Scott A. Rice,^1,4,5^ Cindy Gunawan ^1,6^ **

^1^ The iThree Institute, University of Technology Sydney, NSW 2007, Australia

^2^ School of Pharmacy and Medical Sciences, The University of South Australia, Adelaide, Australia

^3^ Department of Microbiology, Tumor and Cell Biology, Karolinska Institutet, Stockholm, Sweden

^4^ Singapore Centre for Environmental Life Sciences Engineering, Singapore

^5^ School of Biological Sciences, Nanyang Technological University, Singapore

^6^ School of Chemical Engineering, University of New South Wales, Sydney, NSW 2052, Australia


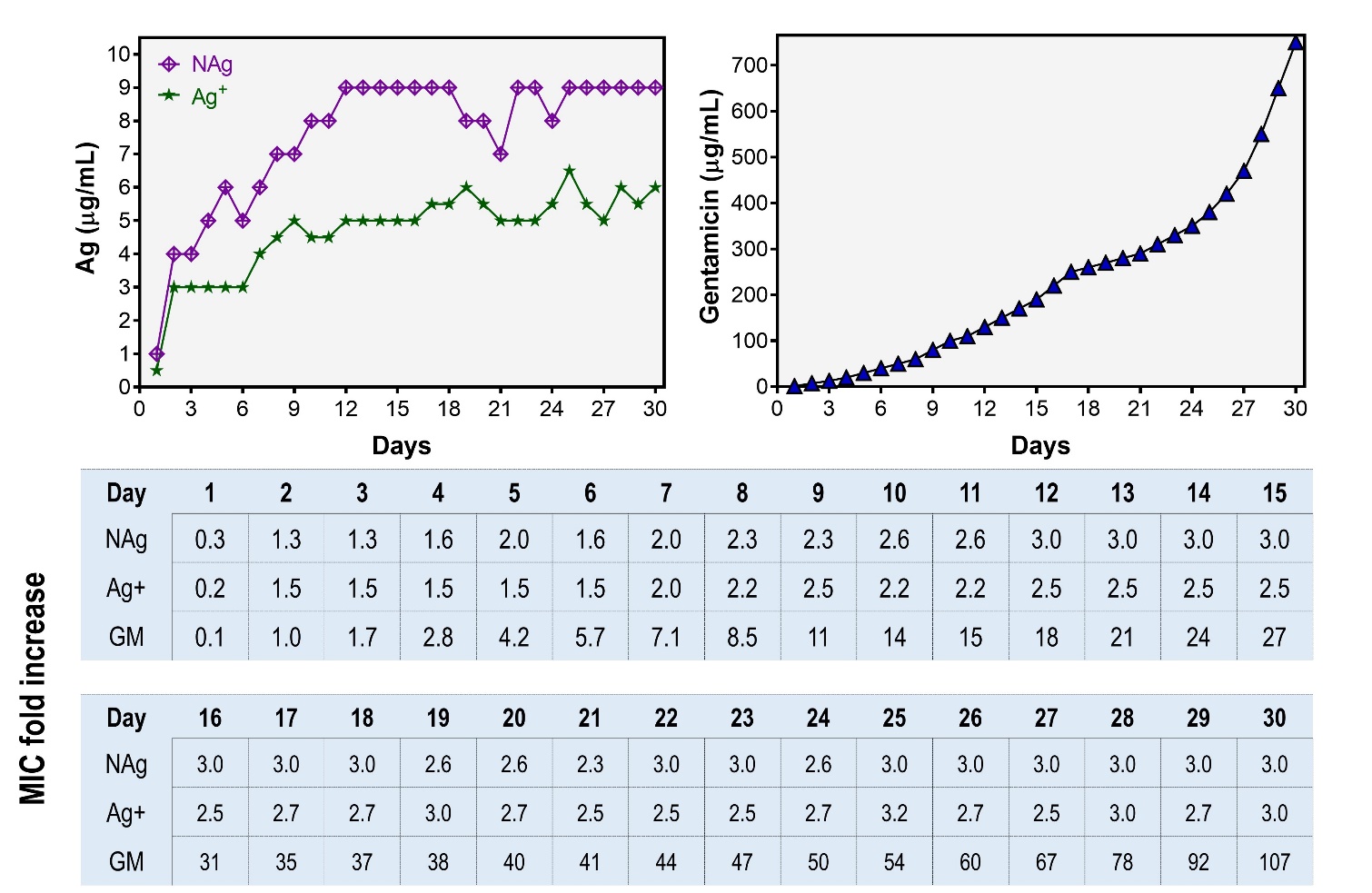


**Figure S1.** The biological replicate of *P. aeruginosa* serial passaging in the presence of progressively increasing concentrations of NAg, ionic silver (Ag^+^) and gentamicin (GM) for 30 d via sub-culturing every 24 h. The passaging experiment also included a cell-only passaged culture (no antibacterial agent). The table details as MIC-fold increase, the shifts in the highest dosage of each agent at which the bacterium could proliferate.


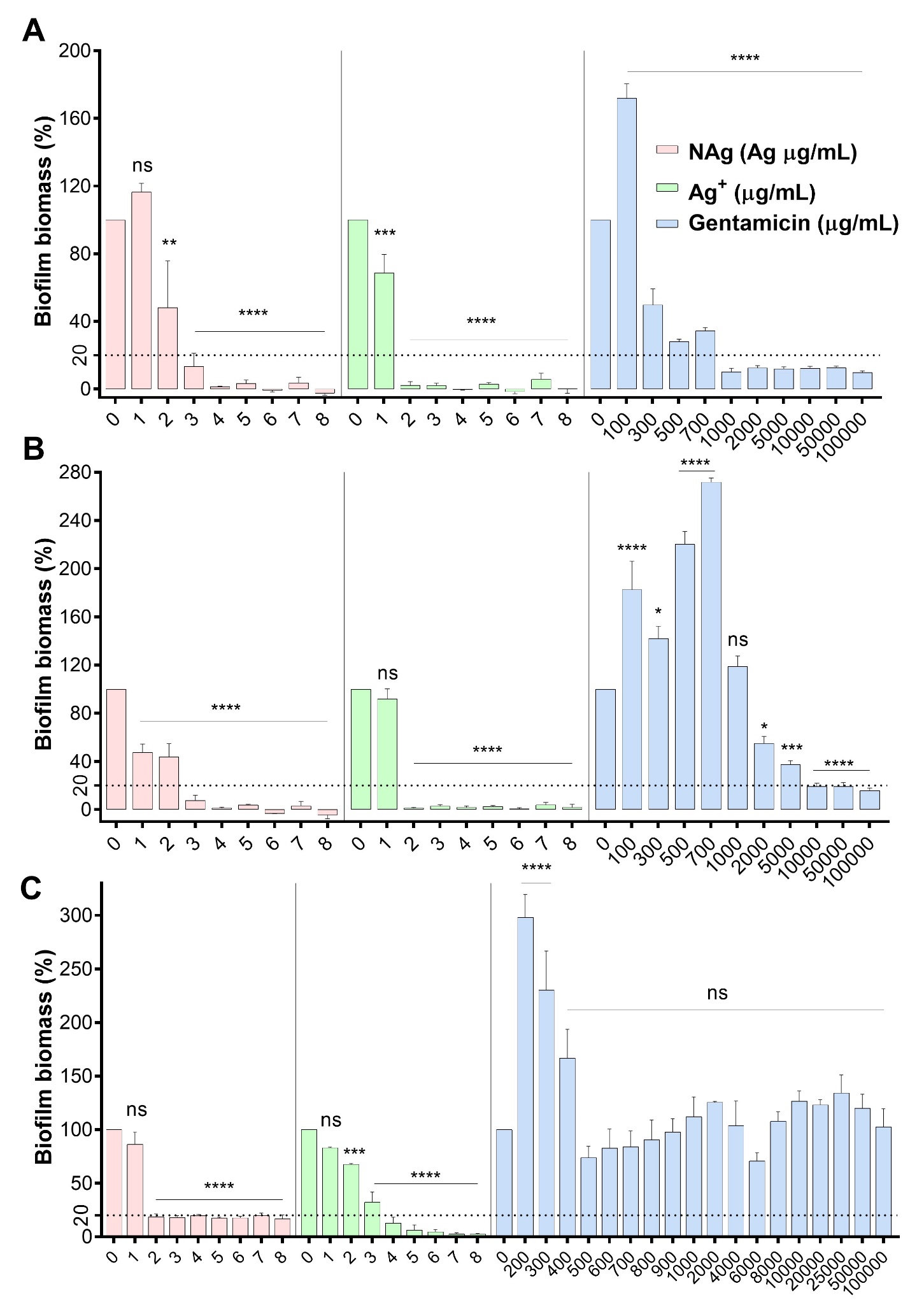


**
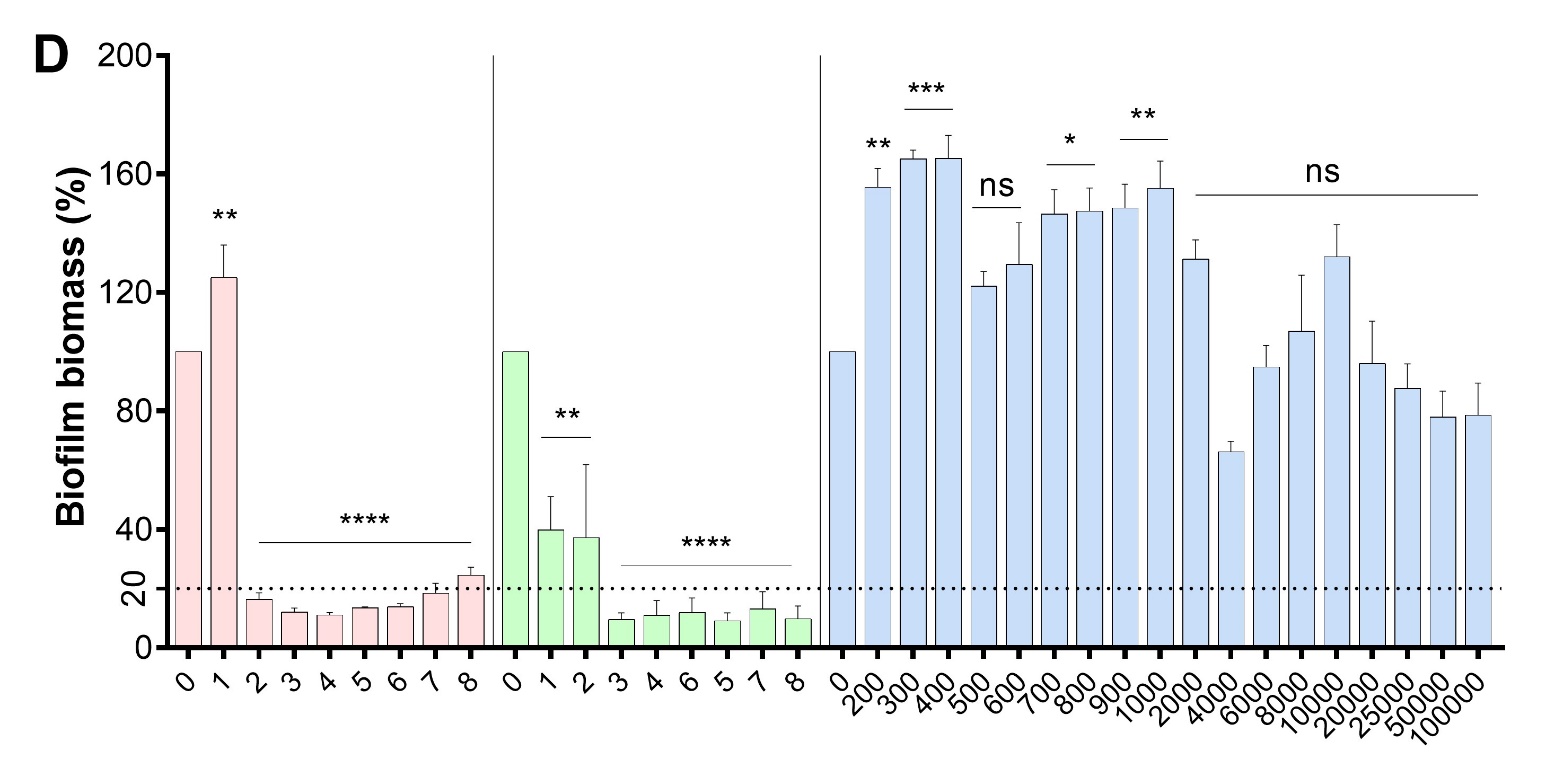
Figure S2.** Post long-term exposure changes in the MBIC (A, B) and MBEC (C, D) of NAg, Ag^+^ and gentamicin (GM) on the respective mid-point passaged (A, C) and end-point passaged (B, D) cultures from the second biological replicate of the passaging experiments. Biofilm biomass is expressed as % relative to the cell-only control (0 µg/mL). Error bars represent SEM (standard error of the mean) of three biological replicates (experiments with independent bacterial inocula from three isolates and different antimicrobial preparations, each with three technical replicates). * indicates statistically significant biofilm growth inhibition (A, B) and eradication of established biomass (C, D) with p > 0.05 (not significant, ns), p < 0.05 (*), p < 0.01 (**), p < 0.001 (***) and p < 0.0001 (****), relative to the cell-only control.


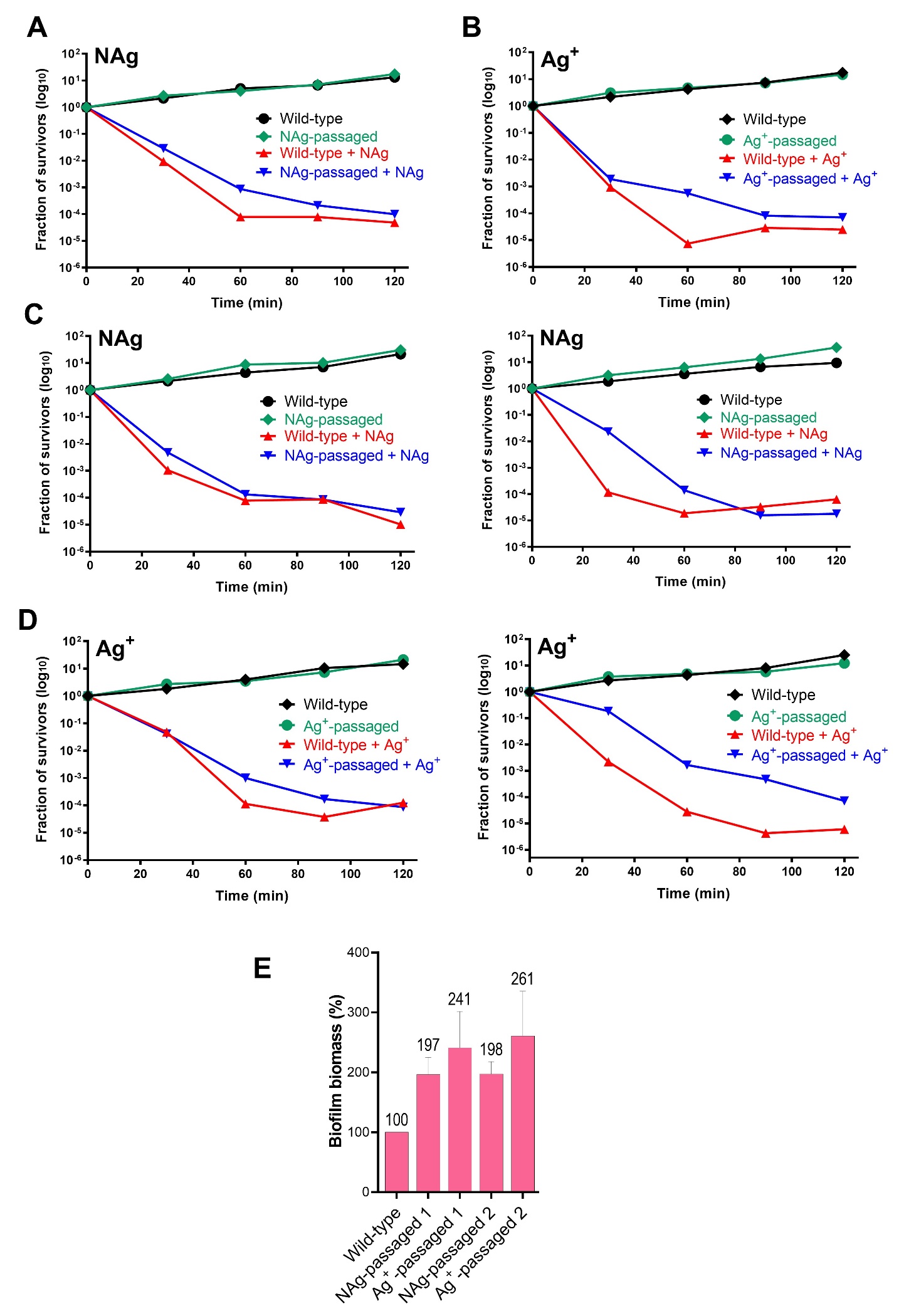


**Figure S3.** The killing kinetics of NAg-passaged and Ag^+^-passaged *P. aeruginosa* in comparison to the wild-type strain. The passaged cultures were from (A, B) the first biological replicate of the passaging experiments; from (C, D) the second biological replicate of the passaging experiments and shown are the two biological replicates of the killing kinetics. The bacterium was exposed to the silver antibacterials at their respective 1.5x MIC dosages (4.5 µg/mL for NAg and 3 µg/mL for Ag^+^). The log_10_ decrease in cell population was determined relative to population at time 0 (as colony forming units). (E) Biomass quantification of biofilms grown from NAg-passaged and Ag^+^-passaged strains in comparison to the wild-type strain.


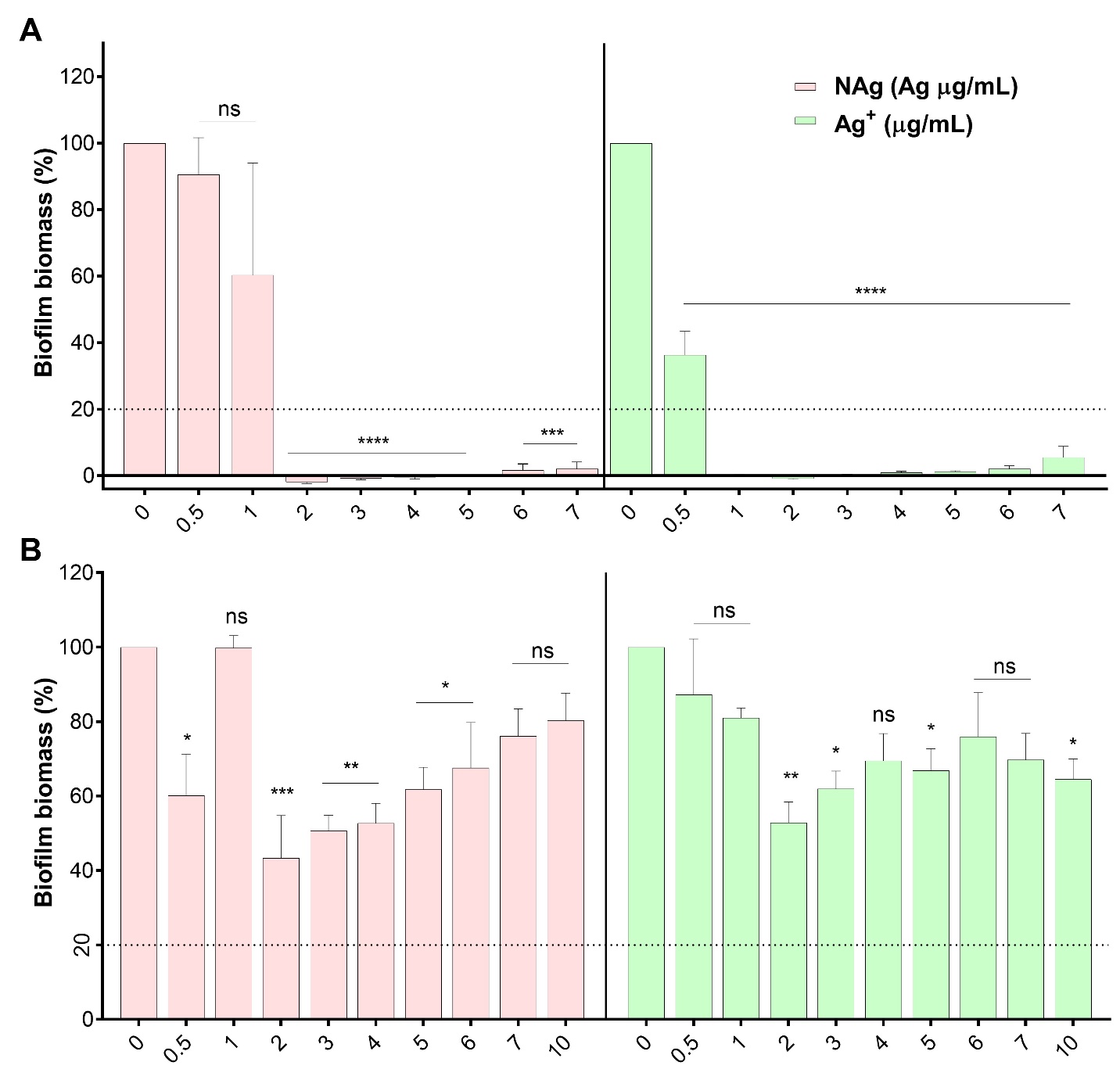


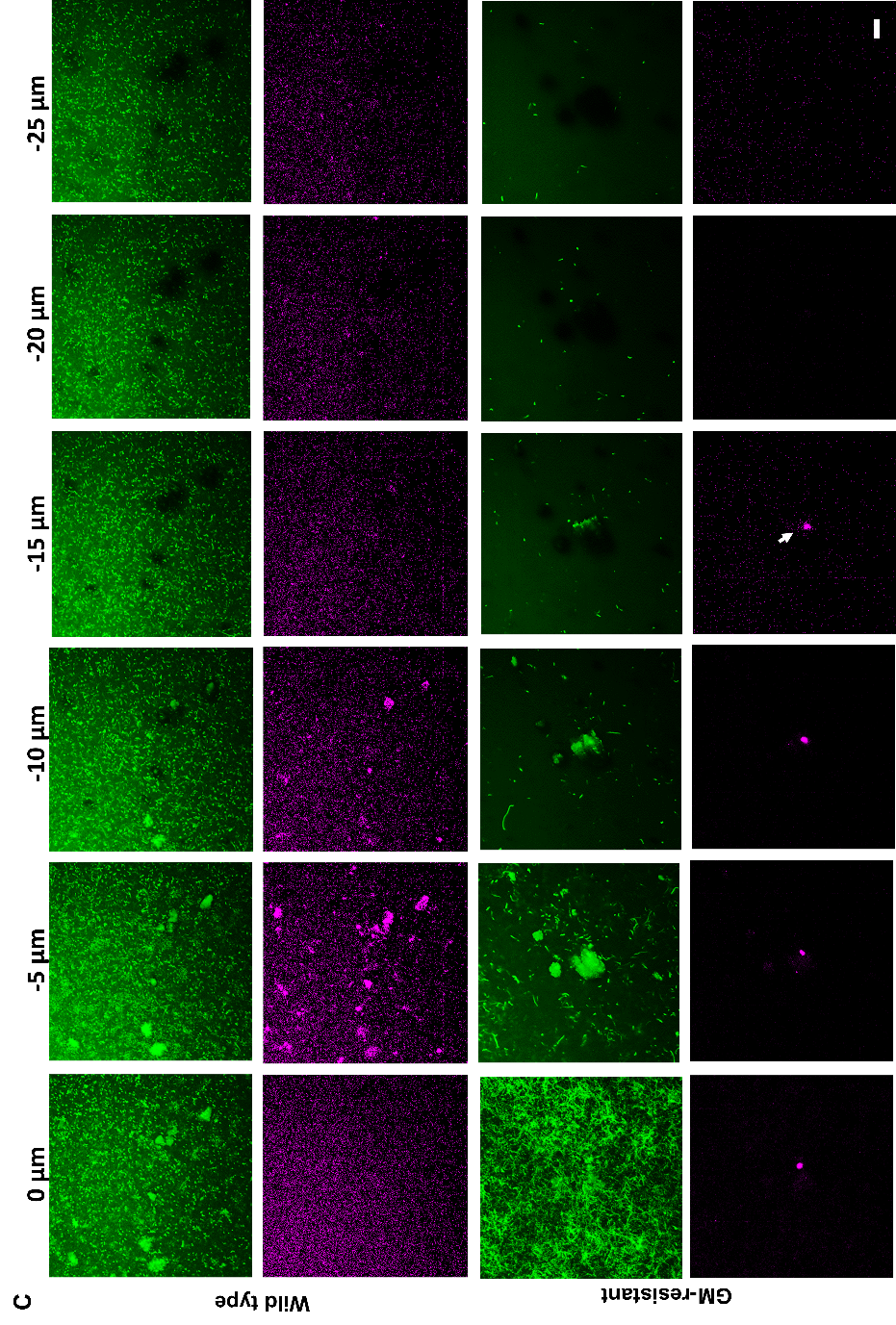


**Figure S4.** (A) Inhibition of biofilm growth of *P. aeruginosa* GM-resistant strain (obtained from the second biological replicate of the passaging experiments) by NAg and Ag^+^ (24 h exposure, 37°C). (B) Eradication of established biofilm of GM-resistant strain (from the second replicate of the passaging experiments) by NAg and Ag^+^ (24 h exposure, 37°C). Biofilm biomass is expressed as % relative to the cell-only control (0 µg/mL). Error bars represent SEM (standard error of the mean) of three biological replicates (experiments with independent bacterial inocula from three isolates and different antimicrobial preparations, each with three technical replicate). * indicates statistically significant inhibition and eradication effects with p > 0.05 (not significant, ns), p < 0.05 (*), p < 0.01 (**), p < 0.001 (***) and p < 0.0001 (****), relative to the cell-only control. (C) Penetration of NAg particle (aggregates) in wild-type and GM-resistant *P. aeruginosa* (obtained from the second replicate of the passaging experiments). Biofilm biomass (green) were exposed to NAg at 20× MIC dosage (3 h, 37°C) and sectional z-stack depth images were acquired using multiphoton microscope. The second harmonic generation and hyper Rayleigh scattering signals of NAg particles (pink) were used to detect their presence in the biofilms. Scale bar = 20 µm
